# The Prognostic Value of Platelet Kinetics Assessment in Pediatric Chronic Idiopathic Thrombocytopenic Purpura

**DOI:** 10.3390/diagnostics15141790

**Published:** 2025-07-16

**Authors:** Nebojsa Igrutinovic, Jelena Pantovic, Bojana Markovic, Marija Medovic, Milica Cekerevac, Vladimir Markovic, Strahinja Odalovic, Sanja Knezevic, Ana Vujic, Isidora Mihajlovic, Nevena Stojadinovic, Dragan Knezevic, Nina Urakovic, Ivana Andrejevic, Gordana J. Ristic, Vladimir Slavkovic, Kristina Andric, Rasa Medovic

**Affiliations:** 1 University Clinical Center Kragujevac, Zmaj Jovina 30, 34000 Kragujevac, Serbia; shone31094@gmail.com (N.I.);; 2 Department of Internal Medicine, Faculty of Medical Sciences, University of Kragujevac, Svetozara Markovića 69, 34000 Kragujevac, Serbia; 3 Center for Nuclear Medicine, University Clinical Center of Serbia, Višegradska 26, 11000 Belgrade, Serbia; drjelena.pantovic@gmail.com (J.P.);; 4 Department of Pediatrics, Faculty of Medical Sciences, University of Kragujevac, Svetozara Markovića 69, 34000 Kragujevac, Serbia; 5 Department of Dermatovenereology, Faculty of Medical Sciences, University of Kragujevac, Svetozara Markovića 69, 34000 Kragujevac, Serbia; 6 Department of Microbiology, Immunology and Fundamentals of Oncology, Faculty of Medical Sciences, University of Kragujevac, Svetozara Markovića 69, 34000 Kragujevac, Serbia; 7 Faculty of Medicine, University of Belgrade, Dr. Subotica Starijeg 8, 11000 Belgrade, Serbia; 8 Department of Communication Skills, Ethics and Psychology, Faculty of Medical Sciences, University of Kragujevac, Svetozara Markovića 69, 34000 Kragujevac, Serbia; 9 Department of Surgery, Faculty of Medical Sciences, University of Kragujevac, Svetozara Markovića 69, 34000 Kragujevac, Serbia; 10 Department of Pathophysiology, Faculty of Medical Sciences, University of Kragujevac, Svetozara Markovića 69, 34000 Kragujevac, Serbia

**Keywords:** chronic idiopathic thrombocytopenic purpura, children, platelet kinetics

## Abstract

**Background/Objectives**: The assessment of platelet kinetics (APK) is recommended for patients with chronic idiopathic thrombocytopenic purpura (chITP). The aim of this study was to examine the importance of APK as a prognostic instrument in the selection of therapy in children with chITP. **Methods:** Retrospective, observational research, which included chITP children who were treated and subjected to APK in Serbia for 25 years (total number was 152). **Results:** In the acute phase of the disease, 15% of patients had life-threatening bleeding, 15% were asymptomatic, and there were no cases of fatal bleeding. Mean platelet life was 0.89 ± 0.47 days. A total of 45% of patients had normal platelet production, and 35% had very low production. Among the patients, 55% exhibited splenic platelet sequestration, 35% had mixed sequestration, and 10% showed hepatic platelet sequestration. Platelet lifespan and production indices were less reliable parameters, due to numerous contradictory results, especially when compared with the location of platelet sequestration. Distribution of bleeding and therapy-resistant patients was dominant with mixed sequestration. Good therapy responders had dominant splenic sequestration. In the chronic phase of the disease, initial therapy was sufficient for 40–45% of patients, while another 25% required second-line therapy, regardless of platelet sequestration location. A total of 25% percent of patients had undergone splenectomy, and all of them were in stable remission. The remaining 10%, which represented the most severe cases, required all available therapies, had equally mixed and liver sequestration, and splenectomy showed no effect. **Conclusions:** APK may be a determining factor for the selection of splenectomy as a therapeutic option in case of predominantly splenic sequestration. Although the platelet production index has been explored in several studies, its clinical relevance remains controversial. In our findings, it did not contribute to therapeutic decision-making and may even lead to misinterpretation. The factors distinguishing the minority of bleeding and therapy-resistant patients with similar laboratory profiles remain unclear.

## 1. Introduction

In 60% of children with newly diagnosed idiopathic thrombocytopenic purpura (ndITP), clinical manifestations may disappear spontaneously or after initial treatment within three months. In another 20–30% they disappear within a year, in which case they are considered a persistent form and require more serious monitoring. Conversely, thrombocytopenia is present for longer than 12 months, and chronic idiopathic thrombocytopenic purpura (chITP) develops in 10–20% of children [1,2].

Approximately 60–70% of patients with chITP require some kind of treatment, while others may have spontaneous recovery. However, factors that can predict which patients will spontaneously recover have not been defined. Moreover, the duration of achieved remission can not be determined in either acute or chronic forms [1,2,3,4].

It is assumed that idiopathic thrombocytopenic purpura (ITP) represents a disease spectrum mostly distinguished by autoantibodies targeted directly against platelet glycoproteins, which cause rapid destruction of circulating platelets [5,6].

Measurements have shown that platelet lifespan is within normal values in certain patients with ITP. This indicates the possibility that autoantibodies may damage megakaryocytes or that only some platelets represent the target for autoantibodies, while they do not react to others. Theoretically, conditions that are distinguished by isolated thrombocytopenia can be caused by other reasons as well, such as poor platelet production due to relative or absolute lack of thrombopoietin [5,6,7].

By observing platelets marked with $51_{Chromium}$ or $111_{Indium}$ isotopes, it was confirmed that 30% of the entire platelet mass was found in the spleen sinusoids and functions as a platelet reserve. Platelets spend one-third of their lifespan in the spleen. Old platelets are largely removed in the red pulp of the spleen. The main pathophysiological process of antiplatelet antibody creation in patients with ITP also occurs in the spleen [8]. So, it is understandable why approximately 70–80% chITP patients respond to splenectomy. To date, there are no reliable preoperative prognostic factors. However, clinicians have observed that a good therapeutic response to reticuloendothelial obstruction is often followed by a favorable response to splenectomy, whereas a good preoperative response to steroids has been associated with inconsistent outcomes [9,10].

The assessment of platelet kinetics is recommended for all patients with ITP who have had a poor response to initial therapy or when the disease progresses to the chronic form. The assessment is based on marking platelets using radiomarkers of exact radioactivity, which is measured according to patients’ body mass, height, and age. Following the application of platelets marked with $51_{Chromium}$ or $111_{Indium}$, platelet lifespan, platelet production index, and location (index) of platelet destruction/sequestration are measured. Data obtained by measuring platelet kinetics may be a determining factor in choosing second-line and third-line therapies, especially when considering splenectomy as a therapy option [11,12,13,14].

Moreover, many authors recommend assessing platelet kinetics as both a prognostic marker and a component of preoperative decision-making regarding splenectomy. A highly significant correlation is particularly noticed between splenectomy results and platelet sequestration location [11,12,14].

On the other hand, some studies consider that preoperative assessment of platelet kinetics should not be conducted because of its inconsistent results and absence of sufficient correlation [13].

All aforementioned studies have been conducted on adult patients. In other studies, the assessment of platelet kinetics has been used to evaluate treatment outcome in patients with ITP [15,16].

The aim of our study was to examine the importance of platelet kinetics assessment in the selection of therapy options and its use as an instrument of prognosis, based on data from a representative number of cases in children suffering from chITP.

## 2. Materials and Methods

### 2.1. Study Design and Participants

This study is designed to be retrospective, observational, and clinical research.

This research included children between the ages of 2 and 18 years diagnosed with chITP who were treated in tertiary pediatric hematology centers in the Republic of Serbia for 25 years and were consecutively enrolled and subjected to platelet kinetics assessment.

Criterion for eligibility: Diagnosed chITP according to guidelines from the American Society of Hematology (Thrombocytopenia that lasts at least 12 months, excluding all other causes of thrombocytopenia) [2].

Criteria for ineligibility: Infants and children younger than 2 years with thrombocytopenia; children with diagnosed or suspected thrombasthenia; children with Evans syndrome; children with chITP and hemostasis disorders; children with secondary chronic thrombocytopenia (liver dysfunction, systemic lupus, and connective tissue disorders).

We used data from the medical history of children with chITP, and the assessment of platelet kinetics conducted at the Center for Nuclear Medicine, University Clinical Center in Belgrade from 1995 to 2020.

We observed basic clinical and laboratory characteristics, and from the parameters included in the assessment of platelet kinetics, we measured mean platelet life, production index, and index (location) of radiolabelled platelet sequestration. Moreover, we analyzed therapy options and treatment outcome, i.e., the degree of achieved remission.

After labeling the platelets with a radioactive marker and determining their count in the complete blood count, the average platelet lifespan was determined from a radioactivity-time curve, obtained by measuring the radioactivity of blood samples collected at 20 min, 2 h, and 4 h after the administration of radiolabeled platelets, and then once daily until the blood radioactivity decreased to approximately 10% of the initial value. A specialized computer program was used to calculate the mean platelet lifespan, and based on this value, along with the platelet count in circulation, the platelet production index was calculated (normal range: 1–2).

To determine the location of platelet sequestration, sequential static scintigrams were obtained in anterior and posterior projections of the patient, ensuring that the liver, spleen, and heart were simultaneously within the field of view of the gamma scintillation camera. These images were subsequently processed using dedicated software. If the calculated ratio was below 0.8, platelet sequestration was considered hepatic; values between 0.8 and 1.4 indicated mixed (hepatic and splenic) sequestration; values from 1.4 to 2 suggested predominantly splenic sequestration; values above 2 indicated exclusively splenic sequestration.

### 2.2. Statistical Data Processing

Mean values ± standard deviation were used for continuous variables, whereas categorical variables were shown in percentages. In order to determine the importance of the difference in frequencies of continuous variables, we used Student’s *t*-test, whereas a chi-square test was used for categorical variables. For comparison between multiple groups, multiple comparison tests were used—specifically, Tukey’s Honestly Significant Difference (HSD) test, or its modification, the Tukey–Kramer test for unequal sample sizes. The value of *p* < 0.05 was considered statistically significant.

## 3. Results

The total number of patients with chITP who met the criteria was 152, with a mild female predominance (F:M = 85:67, i.e., 1.27:1) and predominance of adolescents and school-aged children (approximately 35% in both groups). The severity of the bleeding manifestations was determined according to the International Working Group on ITP [17] and the need for blood derivative transfusion. In the acute phase of the disease, 15% of patients had life-threatening bleeding, 15% were asymptomatic, and there were no cases of fatal bleeding. Blood derivative transfusion was not required for 70% of patients, but 15% required more than ten transfusions for the duration of the disease. Around 75% of patients in the acute phase of the disease had a good response to initial therapy lasting at least 6 weeks or longer, whereas 25% were resistant to initial therapy.

The mean platelet count at diagnosis of chITP was 35.7 ± 9.6 × $10^{9}$/L. 40% of patients had a platelet count <20 × $10^{9}$/L, with only 5% above 50 × $10^{9}$/L. All patients underwent a cytological examination of bone marrow aspirates. Hyperplasia of megakaryocytes was found in only 50% of cases, rare megakaryocytes were observed in 25%, and no patients showed evidence of bone marrow dysplasia.

Mean platelet life in the examined group was 0.89 ± 0.47 days. It is notable that a mean platelet lifespan of less than 24 h was observed in most patients with chITP (around 70%). On the other hand, around 15% had a normal platelet lifespan. In 45% of patients, normal platelet production was measured (index > 2), while 35% had a very low degree of platelet production in the bone marrow (index < 1). In almost 55% of patients, the spleen was either the dominant or the single organ of platelet sequestration, whereas in 35% mixed sequestration was present in the liver and spleen. In 10% of patients, the spleen was not the location of platelet sequestration; that role was taken over dominantly by the liver, lung, stomach, testicles, or thymus.

Around 60% of patients with a mean platelet life of <24 h had an acceptable platelet count of >20 × $10^{9}$/L and increased production of megakaryocytes in the bone marrow. Conversely, 50% of patients with normal platelet lifespan had a platelet count of <20 × $10^{9}$/L and rare megakaryocytes were found in the bone marrow. Most patients with severe bleeding who received many transfusion treatments had a platelet lifespan of <24 h. In the acute phase of the disease, almost 90% of patients with a platelet lifespan of <24 h had a good response to initial therapy, whereas over 40% of patients with close-to-normal values of platelet lifespan were resistant to initial therapy (Table 1).

In approximately 70% of patients with a low production index (<1), platelet count values were <20 × $10^{9}$/L, whereas for 85% of patients with a good production index (>2), platelet count was >20 × $10^{9}$/L. Most patients who had severe bleeding (Bleeding Severity Score (BSS) graded at 3 and 4) and who received multiple transfusions had a low platelet production index (<1). On the other hand, there was no clear correlation between platelet production index, percentage of megakaryocytes in the bone marrow, and initial therapy response in the acute phase of the disease (Table 2).

In around 70% of patients with mixed platelet sequestration, a platelet count of <20 × $10^{9}$/L was measured, whereas a platelet count of >20 × $10^{9}$/L was measured in patients with splenic sequestration (around 80%). Only 15% of patients with dominant splenic sequestration experienced severe bleeding, whereas patients with bleeding predominantly had mixed sequestration. Similar results were obtained regarding transfusion treatments–more than 90% of patients with splenic sequestration either received <5 transfusion treatments or none. Moreover, in the acute phase of the disease, most patients with a good response to initial therapy had platelet sequestration in the spleen, whereas patients with mixed sequestration were resistant. Even in this case, the percentage of megakaryocytes does not indicate a correlation with the location of platelet sequestration (Table 3).

The analysis of patients’ medical histories showed that, after the first 12 months of disease duration, which is the chronic phase of the disease, repeated initial therapy alone was sufficient for approximately 45% of patients. Afterwards, patients entered a stable phase (complete clinical remission). Approximately 20% of patients received initial therapy and available second-line therapy. Most of these patients occasionally had mild signs of hemorrhagic syndrome or became asymptomatic. In the acute phase of the disease, around 25% of patients underwent splenectomy as remaining treatment, and another 10% of patients received this treatment as part of therapy in combination with second-line and third-line therapies. It should be stated that the last group of cases was very severe, and the disease remained active for five years of treatment.

The analysis of the influence of platelet lifespan on therapy selection showed that patients with normal platelet lifespan in the chronic phase of the disease were treated with either initial therapy alone or in combination with second-line therapy. A total of 90% of patients who underwent splenectomy had a mean platelet life of <24 h, and 90% of patients with severe medical conditions had a similar platelet lifespan. For these patients, all available therapy options were required (Table 4).

On the other hand, the results showed no correlation between the platelet production index and the selection of therapy options in patients with chITP.

Regarding the effect of platelet sequestration location in the chronic phase, repeated initial therapy alone was sufficient for about 50% of patients with mixed or liver-dominant sequestration, and for 40% of those with spleen-dominant sequestration. In all patients who underwent splenectomy following repeated initial therapy, the spleen was the dominant organ of platelet sequestration. Patients with severe medical conditions who received all available therapy options had mixed and dominant sequestration in the liver being equally present (Table 5).

Regarding the degree of achieved remission, all patients who received repeated initial therapy, by itself or paired with second-line treatment, and who had undergone splenectomy as a therapeutic option, were in complete clinical and laboratory remission. On the other hand, occasional relapses occurred in patients for whom all available therapy was required, regardless of the performed splenectomy.

## 4. Discussion

Our study of 152 cases of chITP children showed that the female-to-male ratio is ~1.3:1 and that the highest percentage of affected children belongs to adolescents and school-aged children (approximately 35% both), which matches data from the literature.

Although bleeding assessment scales for evaluating bleeding degrees are established [17,18], discrepancies among researchers in this field are still present. The percentage of asymptomatic patients with chITP ranges from 10 to 70% in different episodes [10,19,20]. In this study, we considered patients to be asymptomatic and in complete clinical remission, despite persistent thrombocytopenia following their initial acute bleeding episode. Moreover, in the second year of the duration of the disease, almost 45% of patients had a stable platelet count and were asymptomatic. Mild bleeding was present in approximately 55% of patients; only 15% had severe, at times life-threatening bleeding (BSS of 4), which is consistent with data from the literature [2,10,19,20,21]. According to the aforementioned data, transfusion of concentrated thrombocytes or deplasmatized erythrocytes was not required for approximately 70% of patients. On the other hand, 20% of patients had to be given more than 10 transfusion treatments for the duration of the disease. The percentage of patients in this study was higher than the percentage in the studies of other researchers (between 5 and 15%) [2,10,19,20,21]. The potential unavailability of other therapy options in the late 1990s and early 2000s in our country presumably caused an increase in the number of patients who received transfusions.

Approximately 75% of patients, in the acute phase of the disease, had a good response to initial therapy (achieved long-term clinical or laboratory remission with mild clinical relapse of >6–8 weeks following intravenous immunoglobulin treatment or exclusion of systemic corticosteroids). On the other hand, around 25% of patients were resistant to administered therapy (absence of remission or temporary relapse). However, the results of the researchers who examined the effects of standard initial therapy were similar to the results from this study [1,2,3,4,10,19,20,21].

All the children with chITP underwent a cytological examination of bone marrow aspirate, and no dysplastic changes were found. In only 50% of patients, increased production of megakaryocytes in the bone marrow was noted, which counts as one of the pathognomonic signs of ITP [2,22]. On the other hand, more than 20% of patients had rare megakaryocytes in the bone marrow, which indicates a complex pathophysiological process of chITP. This proves that autoantibodies affect both platelets and megakaryocytes [5,6].

Mean platelet life obtained from the results of the platelet kinetics assessment was around 20 h, which means that most patients had a mean platelet life of <24 h. On the other hand, in around 15% of patients with chITP, close-to-normal values of platelet lifespan were measured (>4 days). This indicates that the pathophysiological pattern of disease onset proves to be very complicated for certain patients. In the case of normal values of platelet lifespan, dysfunction of B-lymphocytes, cytostatic T-lymphocytes, autoantibodies’ influence on megakaryocytes, and partial or complete absence of thrombopoietin may cause weak platelet production and thrombocytopenia [2,5,6,7,23].

However, many authors used to consider platelet lifespan and production index as less reliable parameters, especially in comparison with the location of platelet sequestration. Their studies were based on the evaluation of the therapy success of splenectomy [11,12,14]. Our study included patients diagnosed with chITP with normal, low, or extremely low degrees of platelet production in the bone marrow, with equal distribution. On the other hand, regarding the success of therapy, most authors agree that the platelet sequestration index (location) plays an important role [11,12,13,14]. In our cohort, in more than 50% of patients, the spleen represented the dominant or single organ of platelet sequestration. In 35% of patients, mixed sequestration was equally present in both the liver and spleen. In 10% of patients, the liver was involved in the process of platelet sequestration but not the spleen, whereas in only three patients, platelet sequestration occurred combined in the thymus, stomach, testicles, and/or lungs.

This study concludes that mean platelet life, based on the assessment of platelet kinetics, correlates with the bleeding degree parameter and the number of received transfusion treatments. Most patients with severe bleeding who received many transfusions of blood derivatives belong to the group of patients with a platelet lifespan of <24 h. Surprisingly, no correlation with platelet count or the percentage of megakaryocytes in the bone marrow was found. However, many interesting contradictory results were obtained. In more than 50% of patients with a mean platelet lifespan of less than 24 h, an acceptable platelet count of >20 × $10^{9}$/L and megakaryocyte hyperplasia in the bone marrow were measured. On the other hand, in almost the same values, patients with normal platelet life had platelet counts of <20 × $10^{9}$/L and rare megakaryocytes. This confirms that the pathophysiological pattern in patients with ITP occurs equally in the bone marrow at the level of megakaryocytes and the peripheral blood at the level of platelets [2,5,6,7,24]. The same results were obtained regarding the relationship between platelet lifespan and initial therapy response. In the acute phase of the disease, almost 90% of patients with a platelet lifespan of <24 h had a good response to initial therapy. However, more than 40% of patients with normal platelet lifespan were resistant to initial therapy (Table 1). Similar results were obtained in studies by authors who used the assessment of platelet kinetics as markers of the first-line and second-line therapy success [15,25].

Around 70% of patients with a low production index (<1) had a low platelet count of <20 × $10^{9}$/L, whereas 85% of patients with a good production index (>2) had a platelet count of >20 × $10^{9}$/L. Most patients with severe bleeding (BSS graded at 3 and 4) who received blood transfusion treatments also had a lower platelet production index (<1). These results confirm basic theoretical findings regarding thrombocytopoiesis [22]. However, the issue of a small number of patients who are an exception remains, which indicates to us the pathophysiological complexity of the disease. On the other hand, no clear correlation between the platelet production index, the percentage of megakaryocytes in the bone marrow, and the response to initial therapy was found in the acute phase of the disease (Table 2). Many authors in this field consider the platelet production index to be the least reliable parameter, based on the results of platelet kinetics assessment [11,12,13,14], particularly because of inconsistencies in the interpretation of its values. Furthermore, the findings of our study suggest that the platelet production index may contribute to clinical misinterpretation.

Regarding the location of platelet sequestration, around 70% of patients with mixed platelet sequestration had a platelet count of <20 × $10^{9}$/L. Patients with dominant sequestration in the spleen (around 80%) had a platelet count of 20–50 × $10^{9}$/L. Regarding platelet sequestration in the liver and other organs, patients were equally distributed between those with a platelet count <20 × $10^{9}$/L and those with a platelet count >50 × $10^{9}$/L. Only 15% of patients with dominant platelet sequestration in the spleen had severe bleeding, whereas bleeding manifestations were more prevalent among patients with mixed sequestration (almost 50% of these patients had a BSS graded of 3 or 4). Regarding dominant platelet sequestration in the liver, an equal number of patients experienced severe bleeding or remained asymptomatic. Similar findings were observed in relation to transfusion therapy: more than 90% of patients with dominant splenic sequestration either received no transfusion or fewer than five transfusions, whereas transfusion-receiving patients were predominantly those with mixed sequestration. In the acute phase of the disease, the results show that most patients with a good therapy response had dominant platelet sequestration in the spleen. Patients with mixed sequestration were the most resistant, whereas among patients with dominant platelet sequestration in the liver (and other organs), the number of patients that had a good response to therapy was equal to the number of patients that were resistant to therapy (Table 3).

The aforementioned results show that platelet sequestration location may determine disease prognosis since most patients had splenic sequestration and mild bleeding manifestations, regardless of the platelet count. For some patients who have severe bleeding, splenectomy may be considered as an additional therapy option. On the other hand, 50% of patients with mixed and dominant platelet sequestration in the liver also had mild bleeding manifestations, especially in the case of platelet sequestration in the liver. However, there is a small number of patients with mixed and/or platelet sequestration in the liver with severe bleeding who are resistant to therapy. The reasons behind these results, as well as what differentiates this small group of patients from patients whose results follow the same laboratory pattern of disease, are still not clear.

Many authors recommend assessment of platelet kinetics as part of preoperative decision-making regarding splenectomy as a helpful predictive instrument. Therefore, studies from Serbia, Italy, and Great Britain that included 40, 65, and 91 patients, respectively, indicated a strong correlation between the results of splenectomy and the location of platelet sequestration, whereas platelet lifespan and production index show no correlation with therapy response and relapse rate. Splenectomy results were positive in patients with ITP with splenic sequestration of marked platelets. The results were negative in patients with dominant platelet sequestration in the liver, whereas the results were more negative than positive in patients with mixed sequestration [11,12,14]. On the other hand, a Belgian group of researchers considers that preoperative assessment of platelet kinetics should not be recommended because of its inconsistent results and absence of clear correlation following the examination of 92 patients [13]. It should certainly be emphasized that all the aforementioned studies were conducted on adult patients.

On the other hand, some researchers used the assessment of platelet kinetics to evaluate the results of first-line and second-line therapy response. A study by Houwerzijl et al. shows different results compared with the results from this study. Patients in their study are diagnosed with ITP and reduced platelet production in the bone marrow. These patients had a significantly higher rate of achieved partial and complete remission following Prednizone therapy. They rarely underwent splenectomy compared with the patients with normal or increased platelet production [15]. For instance, Meyer et al. evaluated the effect of Eltrombopag therapy based on the results from the assessment of platelet kinetics in a small number of patients [16]. Nevertheless, new possibilities for the application of the platelet kinetics assessment are yet to emerge since its results remain contradictory.

The results obtained in this study were as expected: in the chronic phase of the disease, either initial or both initial and individual second-line therapies were required for all the patients with normal platelet lifespan (most patients were in stable clinical remission). Taking into consideration that the spleen is the central pathophysiological organ in patients with ITP, it may be expected that 90% of patients who underwent splenectomy following the initial therapy had a mean platelet life of <24 h. The results obtained at different intervals show that all patients (34 of 34) were in stable clinical and laboratory remission. The remaining four patients with a platelet lifespan of 24 to 72 h who had undergone splenectomy following initial therapy were also in stable remission (Table 4). Based on the results from a major study of almost 270 splenectomized patients with chITP, the efficiency of splenectomy is proven to be 96% in patients younger than 30 years of age and 91% in patients older than 30 years (location of platelet destruction was in the spleen alone), whereas splenectomy was an efficient therapy option in only 8% of patients with the location of platelet destruction in the liver or mixed [25].

On the other hand, all available therapy was required for 90% of patients with severe medical conditions. These patients had a platelet lifespan of <24 h, which was an expected result; however, a rich pathophysiological substrate that does not depend on the platelet lifespan alone was found. This may lead to the conclusion that platelet lifespan represents an important prognostic tool, although an additional one. As studies show, including this one [11,12,13,14], the platelet production index points to no correlation with the selection of the therapy option. Platelet sequestration location remains the most important parameter in the assessment of platelet kinetics that affects the selection of therapy for patients with chronic ITP.

The results from the analysis of the influence platelet sequestration location has on the selection of therapy option show that repeated initial therapy alone in the chronic phase of the disease was required for approximately 45% of patients with mixed or dominant platelet sequestration in the liver and 40% of patients with dominant platelet sequestration in the spleen. It should be stated that these patients are in remission in the first 2 years of the duration of the disease. All splenectomized patients with dominant platelet splenic sequestration were in stable remission. Twelve patients with dominant platelet sequestration in the spleen achieved stable clinical remission and varying degrees of laboratory remission. These patients did not undergo splenectomy for various reasons; however, initial and second-line therapy were administered. The remaining twenty patients with mixed platelet sequestration who did not undergo splenectomy received initial and second-line therapy, occasionally showed signs of hemorrhagic syndrome (mild bleeding manifestations or 1–2 episodes of severe bleeding per year), and required therapy. The administration of all available therapy was required for patients in the most severe medical conditions. These patients had equally mixed and dominant platelet sequestration in the liver; however, no patient had dominant platelet sequestration in the spleen. Splenectomy showed no effect on the course of disease, and severe bleeding episodes (≥3) occurred annually (Table 5). Several important limitations of this study must be acknowledged: the long study period of 25 years, during which major changes in therapeutic protocols occurred—particularly concerning second-line treatments and the previously more liberal approach to splenectomy. Furthermore, the management of chronic ITP still largely depends on the individual judgment of the treating pediatric hematologist, which is influenced by numerous factors. Therefore, even minor differences between treatment centers must be taken into account.

## 5. Conclusions

The assessment of platelet kinetics may be a determining factor for disease prognosis and selection of therapy options, especially when the spleen is the dominant organ of platelet sequestration.

Apart from the fact that most patients exhibit mild symptoms regardless of platelet kinetics parameters, for a small group of patients with severe bleeding and dominant splenic sequestration, splenectomy remains the only treatment option.

Conversely, in the cases of patients with mixed and dominant platelet sequestration in the liver, a small number with an identical laboratory pattern of disease have severe bleeding and are resistant to therapy.

Unfortunately, a clear pathophysiological substrate could not be determined in these 10% chITP patients, and the reasons why this entire immunological process occurs and what makes it different from the patients with an identical pattern of disease remain unknown.

While the platelet production index proved clinically unhelpful, platelet lifespan showed a strong correlation with disease severity, offering both practical guidance for physicians and psychological reassurance for patients and families.

What is clearly needed in future research is a well-defined prospective approach to clarify the conflicting findings reported in studies assessing platelet kinetic parameters.

## Figures and Tables

**Table 1:** The influence of the mean platelet life in children with chronic idiopathic thrombocytopenic purpura (chITP) on the studied parameters.

	Mean Platelet Life					
	<1 Day (*N* = 107)		1–4 Days (*N* = 23)		>4 Days (*N* = 22)	
Platelet count (×10^9^/L) *	*N*	%	*N*	%	*N*	%
<20	41	38.3	10	43.5	11	50.0
20–50	64	59.8	11	47.8	6	27.2
>50	2	1.9	2	8.7	5	22.8
Percentage of megakaryocytes *	*N*	%	*N*	%	*N*	%
Rare	17	15.9	7	30.4	10	45.4
Normal	26	24.3	11	47.8	7	31.8
Hyperplasia	64	59.8	5	21.8	5	22.8
Bleeding Severity Score (BSS) #	*N*	%	*N*	%	*N*	%
0–2	67	64.5	16	69.6	20	90.9
3–4	38	35.5	7	30.4	2	9.1
Received transfusion #	*N*	%	*N*	%	*N*	%
<5 times	79	73.8	17	73.9	21	95.4
>5 times	28	26.2	6	26.1	1	4.6
Initial therapy response #	*N*	%	*N*	%	*N*	%
Good response	95	88.8	8	34.8	12	54.5
Resistance	12	11.2	15	65.2	10	45.5

* *p* > 0.05, # *p* < 0.05.

**Table 2:** The influence of the platelet production index in children with chITP on the studied parameters.

	Platelet Production Index					
	<1 (*N* = 56)		1–2 (*N* = 51)		>2 (*N* = 45)	
Platelet count (×10^9^/L) #	*N*	%	*N*	%	*N*	%
<20	38	67.8	15	29.4	7	15.6
20–50	16	28.6	35	68.6	32	71.1
>50	2	3.6	1	2.0	6	13.3
Percentage of megakaryocytes *	*N*	%	*N*	%	*N*	%
Rare	9	16.1	12	23.5	13	28.9
Normal	17	30.3	20	39.2	7	15.5
Hyperplasia	30	53.6	19	37.2	25	55.6
BSS #	*N*	%	*N*	%	*N*	%
0–2	19	33.9	43	84.3	43	95.5
3–4	37	66.1	8	15.7	2	4.5
Received transfusion #	*N*	%	*N*	%	*N*	%
<5 times	31	55.4	43	84.3	43	95.5
>5 times	25	44.6	8	15.7	2	4.5
Initial therapy response *	*N*	%	*N*	%	*N*	%
Good response	41	73.2	39	76.5	35	77.8
Resistance	15	26.8	12	23.5	10	22.2

* *p* > 0.05, # *p* < 0.05.

**Table 3:** The influence of the platelet sequestration index in children with chITP on the studied parameters.

	Platelet Sequestration Index (Location)					
	Liver and Other Organs (*N* = 14)		Mixed (*N* = 56)		Spleen (*N* = 82)	
Platelet count (×10^9^/L) #	*N*	%	*N*	%	*N*	%
<20	8	57.1	38	67.8	16	19.5
20–50	0	0	18	32.1	63	76.8
>50	6	42.9	0	0	3	3.7
Percentage of megakaryocytes *	*N*	%	*N*	%	*N*	%
Rare	9	64.3	13	23.2	12	14.6
Normal	0	0	21	37.5	23	28.1
Hyperplasia	5	35.7	22	39.3	47	57.3
BSS #	*N*	%	*N*	%	*N*	%
0–2	6	42.9	29	51.8	70	85.4
3–4	8	57.1	27	48.2	12	14.6
Received transfusions #	*N*	%	*N*	%	*N*	%
<5 times	6	42.9	35	62.5	76	92.7
>5 times	8	57.1	21	37.5	6	7.3
Initial therapy response *	*N*	%	*N*	%	*N*	%
Good response	6	42.9	37	66.1	72	87.8
Resistance	8	57.1	19	33.9	10	12.2

* *p* > 0.05, # *p* < 0.05.

**Table 4:** The influence of the mean platelet life in children with chITP on applied therapeutic procedures.

	Mean Platelet Life		
	<1 Day (*N* = 107)	1–4 Days (*N* = 23)	>4 Days (*N* = 22)
Therapeutic procedures	*N*	*N*	*N*
Initial therapy (*N* = 65)	37	11	17
Splenectomy (*N* = 38)	34	4	0
Initial + second-line therapy (*N* = 32)	21	6	5
All available therapies (*N* = 17)	15	2	0

**Table 5:** The influence of the platelet sequestration index in children with chITP on applied therapeutic procedures.

	Platelet Sequestration Index (Location)		
	Liver (*N* = 14)	Mixed (*N* = 56)	Spleen (*N* = 82)
Therapeutic procedures	*N*	*N*	*N*
Initial therapy (*N* = 65)	6	27	32
Splenectomy (*N* = 38)	0	0	38
Initial + second-line therapy (*N* = 32)	0	20	12
All available therapy (*N* = 17)	8	9	0

## Data Availability

Raw data supporting the conclusions of this article will be made available by the authors upon reasonable request.

## Abbreviations

The following abbreviations are used in this manuscript:

ITP	Idiopathic thrombocytopenic purpura
ndITP	Newly diagnosed idiopathic thrombocytopenic purpura
chITP	Chronic idiopathic thrombocytopenic purpura
APK	Assessment of platelet kinetics
BSS	Bleeding Severity Score
